# Assessing the feasibility, acceptability and impacts of an education program on hepatitis B testing uptake among ethnic Chinese in Australia: results of a randomised controlled pilot study

**DOI:** 10.1186/s12889-021-11916-0

**Published:** 2021-10-15

**Authors:** Yinzong Xiao, Jack Wallace, Marvad Ahad, Caroline van Gemert, Alexander J. Thompson, Joseph Doyle, Ho Yin Lam, Kico Chan, Gabrielle Bennett, Emily Adamson, Nafisa Yussf, Aurora Tang, Alisa Pedrana, Mark Stoove, Margaret Hellard, Jessica Howell

**Affiliations:** 1 Burnet Institute, Melbourne, Victoria 3004 Australia; 2 Department of Gastroenterology, St Vincent’s Hospital, Fitzroy, Victoria 3065 Australia; 3 University of Melbourne, Parkville, Victoria 3010 Australia; 4 La Trobe University, Bundoora, Victoria 3086 Australia; 5 Centre for Social Research in Health, UNSW Australia, Kensington, New South Wales 2052 Australia; 6 School of Public Health and Preventive Medicine, Monash University, Melbourne, Victoria 3004 Australia; 7 Department of Infectious Diseases, The Alfred and Monash University, Melbourne, Victoria 3004 Australia; 8 Cancer Council Victoria, Melbourne, Victoria 3004 Australia; 9 The Peter Doherty Institute for Infection and Immunity, Melbourne, Victoria 3000 Australia; 10 Hepatitis Victoria, North Melbourne, Victoria 3051 Australia

**Keywords:** HBV testing, Community-based program, Chinese community, Feasibility, Acceptability, Efficacy

## Abstract

**Background:**

In Australia, Chinese migrants are among the populations most affected by hepatitis B virus (HBV) infection but often experience late diagnosis or access to clinical care. This study aims to explore approaches to increase HBV testing in Australia’s Chinese community and inform evaluation planning, specifically to i) assess the feasibility and acceptability of HBV educational programs, and ii) compare HBV testing uptake in people receiving a tailored education resource focussing on liver cancer prevention compared with a standard HBV education package.

**Methods:**

This is a pre-post mixed-methods pilot and feasibility study. People of Chinese ethnicity and unsure of their HBV infection or immunity status were recruited from ten community sites in Melbourne, Australia in 2019–2020. Participants were randomised to receive an education package (comprised of a leaflet and in-person one-on-one educational session) with a focus on either 1) standard HBV-related information, or 2) liver cancer prevention. Participants completed a baseline questionnaire prior to receiving the intervention and were followed up at 6 months’ time for a questionnaire and an opt-in semi-structured interview. Primary study outcomes included feasibility of study procedures, measured by recruitment, participation, and retention rates; acceptability of the education program assessed by acceptability scores; and HBV testing uptake rate in each arm. Secondary outcomes include HBV-related knowledge change, assessed by pre-post comparison; and factors affecting participants’ testing behaviour analysed using qualitative data.

**Results:**

Fifty-four participants received an education package; baseline and follow-up data from 33 (61%) were available. The study procedures of recruitment and retention were feasible; the acceptability of the education program was moderate with improved HBV-related knowledge observed. Four participants self-reported being tested: one (1/15, 7%) in the standard HBV information group and three (3/18, 17%) in the liver cancer prevention information group. Factors identified as affecting testing included perceived relevance and seriousness of HBV, healthcare access and costs of testing, and perceptions of the role of primary care providers in HBV-related care.

**Conclusion:**

A tailored education program targeting ethnic Chinese in Australia was feasible with moderate acceptability. A larger study is required to determine if a liver cancer prevention message would improve HBV testing uptake in Chinese community than standard HBV education message. Supports from healthcare providers, community-based testing programs, and public health education programs are likely needed to motivate diagnostic testing among Chinese people at risk of HBV infection.

**Supplementary Information:**

The online version contains supplementary material available at 10.1186/s12889-021-11916-0.

## Background

Globally, an estimated 296 million people were living with chronic hepatitis B (CHB) in 2019; with around 820,000 deaths per year attributed to complications including liver cancer and liver failure [1, 2]. Despite highly effective treatments, it was estimated that less than 5% of people requiring CHB treatment received antiviral therapy in 2016, with a primary reason being a significant underdiagnosis of the disease; approximately 90% of people with CHB are unaware of their infection status [2, 3]. This level of underdiagnosis highlights the need for interventions to increase hepatitis B awareness, testing and diagnosis.

In Australia, around 1% of the population were estimated to be living with CHB in 2018, among which almost one-third were estimated to be undiagnosed [4], leading to missed opportunities for timely treatment. Australian guidelines recommend testing of hepatitis B virus (HBV) for those at increased risk, with the main risk population being people born in a country with an intermediate or high prevalence of HBV infection [5, 6]. Chinese-born people living in Australia are considered a key at-risk group, comprising around 2.2% of the total Australian population but over 27% of the estimated population living with CHB [7, 8].

Inadequate awareness and knowledge about HBV have been reported as key barriers to testing in Chinese communities, both in Australia and internationally [9–13]. One challenge in providing HBV-related education is ensuring information delivered is both culturally sensitive and motivating. Social psychological studies showed different message framing can affect health behaviour outcomes [14, 15], for example, by emphasising the benefits of adopting a health behaviour versus the costs of not taking a behaviour [16]. Health promotion information for communities most affected by HBV has primarily focused on describing modes of transmission, the virus and its impact on the liver, and its role in causing cirrhosis or liver cancer [17]. This broad educational approach provides considerable information for people to process and usually focusses on the individual cost of neglecting asymptomatic HBV infection. This contrasts with common cancer screening messages used in breast, colon or cervical cancer prevention, where health promotion messages are simpler, focus on getting tested, and emphasise the benefits of early diagnosis [18–20]. Relative to providing general information about HBV, framing HBV testing as a liver cancer prevention strategy may improve peoples’ awareness and increase testing behaviour [21].

Demonstrating the effectiveness of public health programs in driving actual changes in desired health behaviours is vital to maximising the impact of scarce resources and garner support from policymakers. However, assessing behavioural change outcomes following exposure to health promotion programs can be challenging. Several published reports of HBV educational programs show high program reach or increased HBV-related knowledge in targeted populations [22–25]; however, limited behaviour change data, such as HBV testing uptake or other health-related outcomes, are available to assess the impacts of interventions on behaviour change [26, 27]. This data is particularly lacking in Australia.

To inform the development of effective HBV testing programs, we conducted a study evaluating effectiveness of different health promotion messages. In the present pilot study, we sought to assess the feasibility and acceptability of the study procedures, as well as the impact on HBV testing uptake of a liver cancer prevention focussed educational resource package compared with a standard HBV educational resource package, which were the primary aims. Secondary aims included i) evaluation of HBV-related knowledge change before and 6-month after the intervention; and ii) exploration of factors affecting participants’ HBV testing intention and behaviour.

## Methods

### Study design, participants, and sample size

The present study was a pre-post mixed-methods study which was conducted in Melbourne, Australia; with participants enrolled between July–December 2019 and followed up in January–June 2020. Individuals 18 years and over, self-identified as of Chinese ethnicity, and who were unsure of their HBV infection/immunity status were eligible to participate. People self-reporting vaccination against HBV or previous HBV testing were excluded. As a pilot study, the intended sample size was governed primarily by logistics and funding, not by effect size and power estimations to determine differences in rates of HBV testing uptake. We aimed to recruit 50 participants to assess feasibility and acceptability as key outcomes; this sample size would allow the detection of a 30% difference in HBV testing uptake between the two study arms, with 80% power and alpha error of 0.05 [28]. Ethics approval was obtained from the Alfred Hospital Ethics Committee (147/19) and Cancer Council Ethics Committee (HREC 1903). All methods were performed in accordance with the relevant guidelines and regulations.

### Study sites and recruitment

Participants were recruited from ten sites located in the greater Melbourne area, an area home to 356,324 individuals of Chinese ancestry in 2016 [29]. These sites were community-based organisations, who after being contacted by the research team, agreed to support recruitment, and Chinese community activity groups (such as senior citizens group, book clubs), health clinics, gyms, and community festivals (such as cancer awareness day event) (**Table s1**). The study was promoted using information posters, didactic information sessions at community sites by multilingual fieldworkers, or study flyer distribution, depending on the recruitment site, up to two weeks prior to recruitment. The study was also advertised online through university student noticeboard, WeChat and a Chinese language radio broadcast in Melbourne.

Individuals were screened for eligibility 1) when attending the study sites on scheduled recruitment day(s), 2) when having completed an expression of interest form, or 3) when contacting the study investigator after receiving study information. Eligible individuals were consented by a fieldworker and enrolled in the study face-to-face at study sites or at agreed public places if recruited online. All study information was delivered in English, Mandarin or Cantonese by bilingual fieldworkers.

### Interventions

Participants were randomised to receive either standard HBV information in Arm 1 or liver cancer prevention information in Arm 2, in the format of a one-page double-sided information sheet (**Figure s1**) and a 15 min in-person, oral educational session delivered one-to-one for all participants. The educational session was tailored to the written resource assigned to each group and contained a Q&A session to address participants questions.

Resources in both research arms were developed by the research team composed of gastroenterologists, infectious disease physicians, nurses, and public health experts, following a standard checklist for assessing written consumer health information [30]. Both information sheets were available in English or simplified Chinese with the contents tailored to Australia’s Chinese community, translated by certified translators, and assessed for cultural appropriateness with representatives of the Chinese community, and back translated to English. Resource readability was designed to be below grade-six level [31]. In-person education sessions were delivered in Mandarin, Cantonese, or English as per participant’s preference by one of the trained fieldworkers.

### Study procedures: randomisation, allocation, and concealment mechanism

Participants were randomised to one of two study arms by block randomisation (block size of 4) in a 1:1 allocation ratio using a computer-generated randomisation list. Group allocation was determined sequentially by sealed opaque envelopes containing the random allocation sequence. Fieldworkers delivering the intervention and participants were aware of group allocation; single-blinded analysis for primary outcome was performed.

### Outcome measures

Prior to receiving the intervention, participants completed a baseline questionnaire administered on a tablet by a fieldworker including a demographic form and general practitioner (GP) details (if any), three questions (two open-ended and one close-ended) assessing knowledge of HBV transmission, symptoms, and treatment availability (**Table s2**). Six months after participation, individuals were followed up by phone. Participants were considered lost to follow-up after three contact attempts by phone calls were made on different days and times and one text message. A follow-up questionnaire was administered, collecting data on testing uptake and intention, GP consultation history, perceived barriers to testing (open-ended questions), feedback on the program (mixed-method measures by rating the clarity and relevance of the information leaflet and in-person oral educational session, respectively, using a Likert scale; as well as free comments) (**Table s5**), and repeated the three questions assessing HBV-related knowledge (**Table s2**). Self-reported HBV testing uptake was validated through contacting the participants’ GP with participant permission. Participants were invited to an opt-in 15–30-min semi-structured interview at follow-up seeking information on understanding of HBV, testing processes, testing intention, testing barriers, and feedback on the education package. The interview guide is included in **Table s3**. All follow-up questionnaires and interviews were administered by phone due to social distancing restrictions as a result of the COVID-19 pandemic during the follow up period.

Primary outcomes included:

1. Feasibility of study procedures measured by recruitment capability (defined as number of sites and visits, time spent, number of people recruited, and recruitment rate), heterogeneous sample characteristics (defined as pattens of participants demographic characteristics), and retention rate;
2. Intervention acceptability measured by rating and feedback obtained at follow-up; and
3. HBV testing uptake in each research arm measured by testing history reported at follow-up;

Secondary outcomes included:

1. HBV-related knowledge change in each research arm measured by pre-and post-intervention comparison of the responses to the knowledge-related questions;
2. HBV testing intention and possible factors affecting testing behaviour/intention using semi-structured interviews and open text comments obtained at follow-up.

### Statistical analysis

Baseline and follow-up questionnaires were administered via REDCap (version 10.5.2, Nashville, USA) in English, Cantonese or Mandarin as per participant preference. Questionnaire data were exported to Excel 2010 (Microsoft, Seattle, USA) and Stata 13.1 (Texas, USA) for quantitative data analysis. Descriptive data were generated for demographic variables and background characteristics including age, sex, birthplace, preferred language, years living in Australia, educational level, types of health insurance. Chi-square tests were used to analyse differences of each categorical variable between groups. Non-response bias was assessed by comparing the demographic measurement of participants who completed, and those who did not complete follow-up, using baseline data. Responses to open-ended knowledge-related questions were analysed using frequencies of keywords in responses, with percentage change of each response keyword compared between two research arms.

Telephone qualitative interviews were electronically recorded with consent. All recordings were transcribed verbatim and translated into English. Interview transcripts and open-text data collected from questionnaires were imported to NVivo 12 Plus (QSR International, Melbourne, Australia) for coding and theme generation. Qualitative data was analysed following thematic analysis steps [32]; all transcripts were coded by YX and discussed with JW, MA and JH, with an agreed analytical framework containing grouped codes developed and subsequently applied to all transcripts. The study was reported following the CONSORT statement for reporting feasibility studies (**Table s8-s9**) [33, 34]. A COREQ checklist [35] guided qualitative study design, analysis, and report (**Table s10**).

## Results

Fifty-four participants were recruited at baseline, with thirty-three (61%) followed-up for data collection at six months. Table 1 shows demographics of participants completing baseline and follow-up questionnaires, the median age of participants at baseline and those who completed follow-up was 33 and 29, respectively**.** Among participants retained to follow-up, approximately two-thirds were female, and most were aged 18–29 years or 60–90 years, had a tertiary under-graduate of post-graduate degree, were born in mainland China, preferred Mandarin and had resided in Australia for 10 years or more. There were no significant differences in participant characteristics between those who completed and lost to follow-up or between study Arm 1 and Arm 2 (**Table s4**). Ten participants who completed follow-up questionnaires consented to complete qualitative interviews (Table 2).

**Table 1:** Demographics of participants in the study, at baseline and follow-up

Variables	Baseline (***n*** = 54)	Completed follow up (***n*** = 33)
Sex, n (%)		
Female	38 (69%)	22 (67%)
Age group, years, n (%)	(*n* = 53)	(*n* = 33)
18–29	25 (47%)	17 (52%)
30–59	11 (21%)	5 (15%)
60–90	17 (32%)	11 (33%)
Birthplace, n (%)		
Mainland China	29 (54%)	19 (58%)
Malaysia	8 (15%)	5 (15%)
Hong Kong	4 (7%)	1 (3%)
Australia	4 (7%)	2 (6%)
Others (including Singapore, Vietnam, Philippines, Taiwan, East-Timor, New Zealand)	9 (17%)	6 (18%)
Preferred language, n (%)		
Mandarin	28 (52%)	18 (55%)
English	16 (30%)	10 (30%)
Cantonese	10 (19%)	5 (15%)
Years been living in Australia, n (%)	(*n* = 53)	(*n* = 32)
Less than 5 year	20 (38%)	13 (41%)
5–10 years (incl. 5 years)	1 (2%)	1 (3%)
10–20 years (incl. 10 years)	14 (26%)	8 (25%)
20 years and over	18 (34%)	10 (31%)
Main health insurance		
Medicare	34 (63%)	20 (61%)
Overseas Student Health Coverage	18 (33%)	12 (36%)
Other private health insurance	2 (4%)	1 (2%)
Level of highest/current education		
Primary	6 (11%)	4 (12%)
Secondary	4 (7%)	2 (6%)
Tertiary	24 (44%)	13 (39%)
Postgraduate	20 (37%)	14 (42%)

**Table 2:** Summary of participants of qualitative interview

Participant	Age	Sex	Group	Visited a doctor in last 6 months	Discussed hepatitis B with a doctor	Test intention (self-report)	Test uptake (self-report)
**1**	45–54	Male	liver cancer prevention info	No	/	No	No
**2**	18–24	Female	liver cancer prevention info	No	/	No	No
**3**	18–24	Female	liver cancer prevention info	Yes	No	Yes	No
**4**	18–24	Male	liver cancer prevention info	Yes	Yes	Yes	Yes
**5**	18–24	Male	liver cancer prevention info	No	/	Yes	No
**6**	18–24	Female	hepatitis B info	No	/	Yes	No
**7**	18–24	Female	hepatitis B info	No	/	Yes	No
**8**	25–34	Male	hepatitis B info	No	/	No	No
**9**	18–24	Male	hepatitis B info	No	/	No	No
**10**	18–24	Male	hepatitis B info	Yes	No	No	No

### Feasibility and acceptability of study procedures and education program

**Fig. 1:**
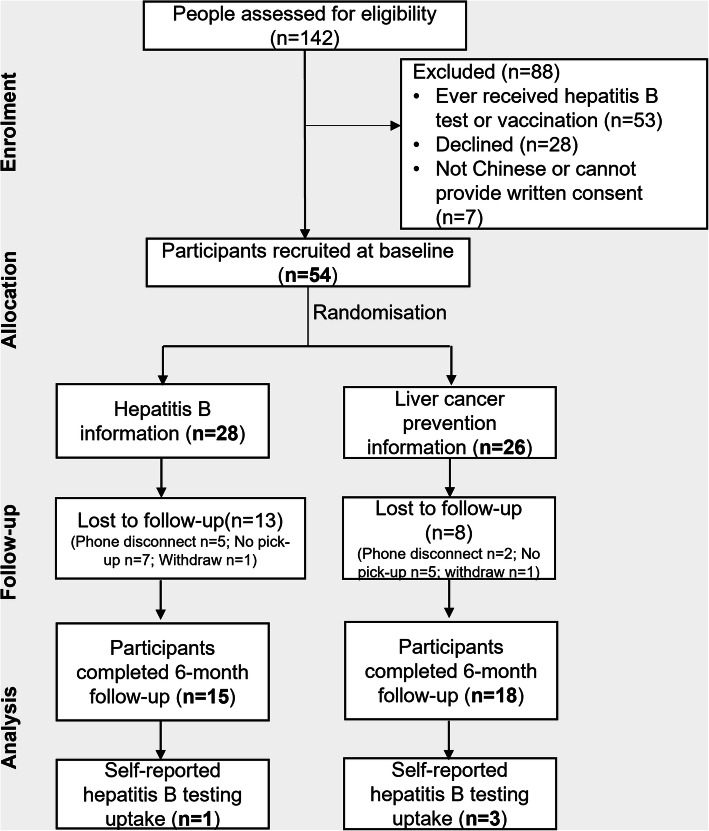
Flow diagram of individuals included in a randomised controlled pilot trial of assessing impact on hepatitis B testing of standard hepatitis B information focused educational package versus liver cancer prevention centred educational package in a Chinese community, Australia, 2019–2020

Nineteen recruitment sessions at ten recruitment sites were completed from July to December 2019; 142 people was assessed for eligibility, among whom 54 participants were recruited. Of the 88 people being excluded, 53 reported having been tested or vaccinated against HBV, 7 were excluded as not being Chinese or cannot provide written, and another 28 people declined the invitation of participation (Fig. 1**,** **Table s1**). At baseline, 20 of the 54 participants did not provide GP (clinic) information, reporting either privacy concerns or not having a regular GP. Follow-up rate at 6 months was 61%; the main reasons for loss to follow-up (*n* = 21) included not answering phone calls (*n* = 12) and phone disconnection (*n* = 7). GP confirmation of HBV testing uptake was not available for three patients who completed the study: two participants withdrew consent to follow up testing history with their GP and one GP clinic refused to provide HBV test details despite the participant’s informed consent.

In terms of acceptability, a median score of 5 (out of 5) was reported in both arms for both leaflet readability and clarity of in-person explanation session; however a higher median score of perceived leaflet relevance was reported in participants receiving the standard HBV information (median score being 4 out of 5) compared to those who in liver cancer prevention information group (median score being 3 out of 5) (**Table s5**). Qualitative interview data identified several positive aspects of information leaflets including simple format, right amount of information, plain language used, clear and well-organised sections, translated version being available, and reputable links provided. Negative aspects of information leaflets identified by interviewees included a lack of pictorial information, information being too basic, and a lack of information about where to get tested. A few participants suggested a shorter follow-up period could improve the recall accuracy as the six-months interval was difficult to provide feedback on the details of resources and study procedures.

### HBV testing uptake and knowledge change

**Fig. 2:**
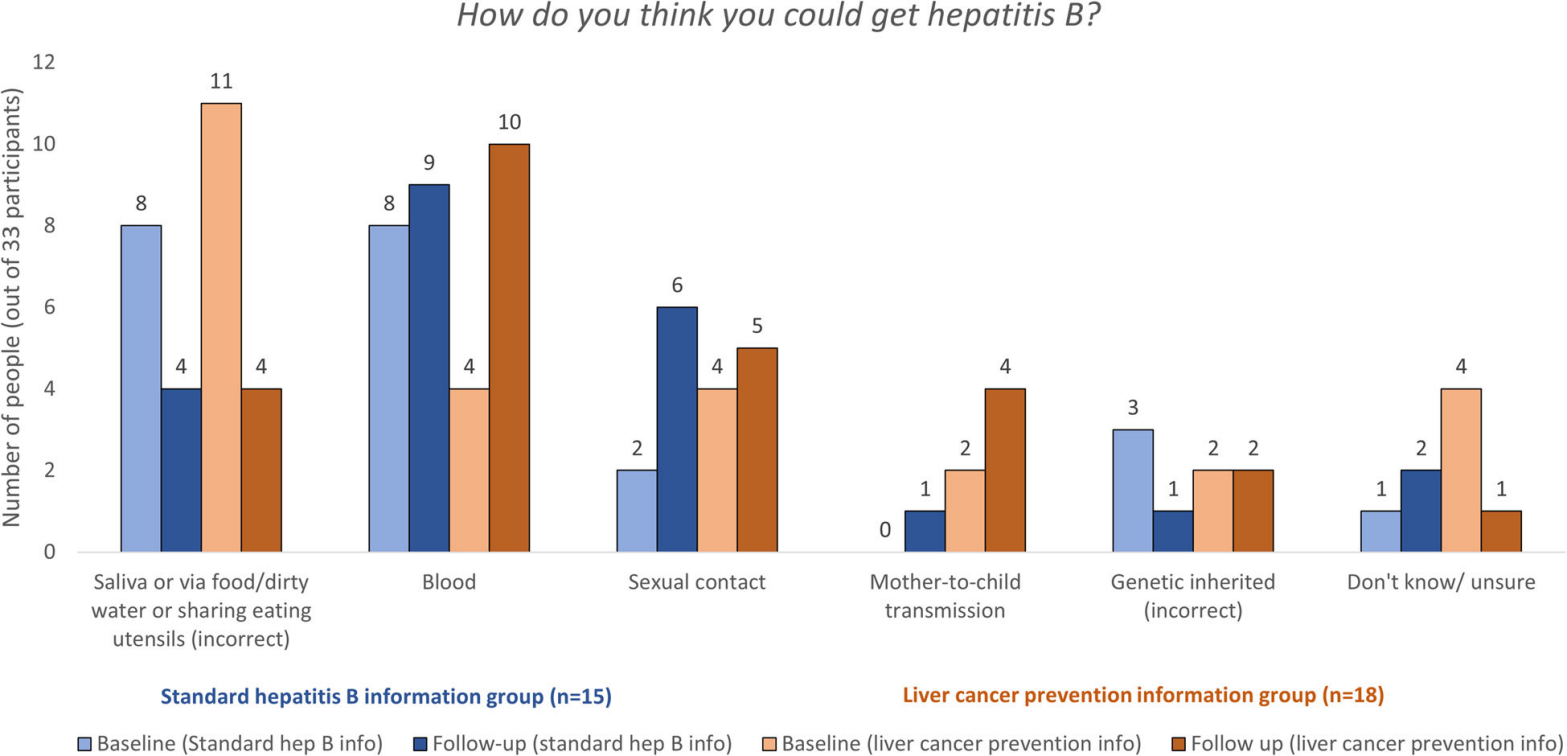
Responses to “how do you think you could get hepatitis B” at baseline and follow-up among participants

At follow-up, four of thirty-three participants (12%) self-reported being tested for HBV; one (1/15, 7%) in the standard information group and three (3/18, 17%) in the liver cancer prevention information group (Fig. 1). HBV-related knowledge change measured by responses to the question “*how do you think you could get hepatitis B*” pre- and post-intervention showed an increased number of participants identified transmission routes correctly, and the number of people who had the misconception that HBV is transmitted through sharing food or eating utensils dropped from 19 (58%) to 8 (24%) (Fig. 2). Knowledge changes pre-and post-intervention in each group showed a similar trend (**Table s6**).

### HBV testing intentions and associated factors

Of the 33 participants who completed follow-up, 21 participants (64%) reported having visited a doctor within six-months of study participation (Table 3). Seven of these participants (that is, 33% of those who visited a doctor) reported discussing HBV testing with their GP (with four receiving a test as reported above). The three who did not have a test reported the “*doctor said I don’t have the problem*” or the “*doctor said I had been tested before*”. Of note 14 of the 21 participants who visited a GP reported not discussing HBV testing. Among the 12 participants who did not visit a GP during study follow-up period, five reported having no intention to be tested (Table 2).

**Table 3:** Consultation history with a doctor and hepatitis B testing intention

	Participants received hepatitis B centred message (*n* = 15)	Participants received liver cancer prevention message (*n* = 18)	Overall (*n* = 33)
Visited a doctor in last six months	9 (60%) ^a^	12 (67%) ^a^	21 (64%) ^a^
Discussed about hepatitis B testing and get tested	1 (11%) ^b^	3 (25%) ^b^	4 (19%) ^b^
Discussed about hepatitis B testing but didn’t get tested	3 (33%) ^b^	0	3 (14%) ^b^
Didn’t discuss hepatitis B testing	5 (56%) ^b^	9 (75%) ^b^	14 (67%) ^b^
Didn’t visit a doctor in last six months	6 (40%) ^a^	6 (33%) ^a^	12 (36%) ^a^
Thought about having a hepatitis B test but didn’t get tested	3 (50%) ^c^	3 (50%) ^c^	6 (50%) ^c^
No intention to have a hepatitis B test	2 (33%) ^c^	3 (50%) ^c^	5 (42%) ^c^

^a^ Among all participants completed follow-up, that is, denominator was the total number of participants in each group or overall

^b^ Among participants visited a doctor in last six months in each group or overall

^c^ Among participants didn’t visit a doctor in last six months in each group or overall

The following factors, drawn from interview and open-text follow-up questionnaire data, were identified as explanations of participant intentions and testing behaviour, including perceived relevance and seriousness of HBV, healthcare access and costs, perceived GP role, and other environmental barriers such as COVID-19 related social restrictions.

#### Perceived relevance and seriousness of CHB

Several participants noted they did not perceive being at risk of HBV infection. Responses such as, “*I don’t think I have the problem*”, were the most common reason for not getting or wanting a test following exposure to the education program. However, two participants later confirmed that they had been vaccinated against HBV after clarification with family members. Several others explained being at low risk for HBV as a result of no family history, not experiencing symptoms, no behaviours such as unsafe sex or injecting, no concerns identified during clinical check-ups, living in big cities, and the belief that a good lifestyle and eating habits could avoid the risk of infection.

*No (intention to get a test). I don’t think I’m showing symptoms. (Participant 10)*

*In addition to the mandatory health examinations (required for visa application), I also sought that kind of screening tests at my own expense…I didn't notice (anything particular from health report) …also…I don’t have bad living habits or intravenous drug use or blood transfusion… nothing. (Participant 5)*

*No, not really (considered getting a test). I don’t think I have a history of hep B, of what my parents have informed me. (Participant 9)*

Five participants noted that they would only get an HBV test if they considered themselves at increased risk, which they understood as being *“if I start showing symptoms” or “if someone that I knew had it”.*

*I think (I would get tested) only if I had notification from a close family member or, that they’ve been tested for hep B and they’re positive…or if we have some family history then I would go. (Participant 7)*

*Probably if I find that I’m experiencing symptoms, uh [laughs] yeah something like that or if I did the general screening and told me I had a high chance of getting it then I would get tested. (Participant 8)*

In addition to being at low risk for HBV infection, low perceived seriousness of HBV was described by two participants.

*I don’t think people … think it’s a serious issue, an urgent issue. [pause] so, like, I don’t want to bother. (Participant 1)*

#### Healthcare access, cost and understanding of the GP role

At follow-up, several participants, all in the 18–29 years age group (**Table s7**), reported being unsure of where they could be tested or only had a vague idea of “*normally you go to the hospital*” or “*probably google and check the nearby clinics and hospitals that provide tests*”. Even though most participants acknowledged getting a HBV test can be relatively easy by “*just go[ing] to see a GP*”, several were concerned of the cost of testing.

*In Australia under my student healthcare, I’d probably ask my GP how much it costs... Or when I’m back home in my own country, I’d just head over to the government hospital and get tested there. (Participant 9)*

*I'm not very sure if I go directly to the small clinic and find a GP, for example, if I say I want to do it, he would ask...for example, I will have to pay extra, or say I have to wait and stuff. (Participant 5)*

*(What stopped you from considering an HBV test?) Probably the cost. (Participant 8)*

Among participants who reported visiting a GP since receiving the education but not discussing HBV testing, two main response types were observed. One was the participant being a passive recipient of healthcare with their family doctor, and usually access healthcare services with a specific provider either *for regular check-up* or for other chronic condition management. While accessing healthcare is not a barrier for HBV testing per se, the fact that HBV testing is not included in regular check-up and that HBV testing could be less prioritised if other established chronic illnesses, led to missed opportunities of getting tested.

*I saw the doctor only because I ran out of medication. Usually, I don't actively ask for any tests from doctors, I'll do what doctor asks me to test. Also, my doctor gave blood slip to me (with my last visit), I haven't gone to the pathologist, I'm not sure what blood tests it includes. (Participant 16, follow-up questionnaire)*

*I have many other diseases, age-related macular degeneration, diabetes, and other diseases so I don't have extra energy to think about this. (Participant 17, follow-up questionnaire)*

A different perspective of the GP role was described by other participants, who would only visit a GP in response to a specific problem, with testing usually *not something that was in mind*:

*I only went to GP for tetanus vaccine due to hand cut. (Participant 3)*

*Only went to GP for allergy problem; didn't come into mind about hep B testing. (Participant 15, follow-up questionnaire)*

Both perspectives led to the same outcome; individuals felt unable to request an “irrelevant” test.

#### Contextual factors

After March 2020, participants were exposed to a jurisdiction-specific state of emergency in response to the COVID-19 pandemic, and which overlapped the follow-up period for the study between January and July 2020. Several participants who considered getting a HBV test minimised low priority activity due to concerns of COVID-19 and/or social distancing restrictions:

*…just the current COVID situation, staying at home. I also have a father who has disability, so it’ll be, I guess, risky for me to go out a lot—especially getting into contact with people in a healthcare, clinical setting. (Participant 7)*

Another participant however noted the COVID-19 pandemic supported their intention to increase control over their health:

*Before COVID-19 pandemic I didn't consider doing the tests; but I'm thinking to have a whole set of testing to know my health. (Participant 16, follow-up questionnaire)*

One participant suggested a free, easy-to-access testing program and described a clear role for HBV testing in liver cancer prevention would facilitate community engagement for testing:

*If I don't have any symptoms, at my own cost, I wouldn't do that (testing). So for example when we reach 50, the government sent us a letter about bowel (cancer) screening, so it's a free faecal test. All I need to do is to send the faecal sample back. totally free. Then I would do that, because it's simple.* (*Participant 13, follow-up questionnaire*).

Another participant, tested through a health service following study participation, was satisfied that the test was *free and very easy*. The participant reported being tested as part of general testing for sexual transmitted infections and noted that regularly seeing the health service advertisement was motivation for HBV testing.

## Discussion

This pilot randomised controlled study examined the feasibility, acceptability, and impact on HBV testing of a community-based education program using liver cancer prevention centred resources compared to standard HBV information resources. It also sought to explore factors affecting testing intentions and inform future programs to increase HBV testing in the Chinese community in Australia. Key findings were that: it was feasible to recruit and follow up participants from Chinese community for a community-based educational interventional study; the education programs were moderately acceptable; both interventions resulted in improved HBV-related knowledge; a higher HBV testing uptake rate was reported in the liver cancer prevention information group in this pilot however overall testing uptake was suboptimal; several factors were identified for future intervention planning, including overcoming perceived low risk of HBV infection, unclear knowledge of testing access and perceptions and realities around costs and patient-doctor relationships, and COVID-19 restricting health seeking activities.

This pilot study demonstrated that our education program and evaluation procedures were feasible and acceptable. In six-month recruitment period, the study team engaged multiple community-based organisations in represented areas of Chinese community; the number of recruitment session conducted and number of people recruited were reasonable given the small scale of the present study; the recruitment rate was comparable with other health promotion interventional studies recruiting participants from community settings [36, 37]. However, a limitation of present study was that the number of people approached was hard to estimate at several sites due to combined recruitment strategies. Several culturally relevant components contributed to recruitment, including collaborating with reputable local health promotion organisations, building trusting relationships with community partners, developing resources with community representatives, and employing bilingual fieldworkers from targeting community [37, 38]. Importantly, one challenge identified by fieldworkers was that while health-related education activities were welcomed in the community, people were unwilling to join the study due to confidentiality concerns, and for participants who consented to join, there was also a concern of disclosing personal information such as date of birth, phone number and their GP. A spread in sex, birthplace, preferred language, and years living in Australia was found among participants, however participants recruited were mostly seniors and young adults, suggesting a challenge recruiting middle-aged people with current study procedures, even though recruitment was conducted in sites where different age group could be captured such as immunisation sessions, reading clubs, and study was promoted via social media. While previous studies showed home visits by lay health workers can engage Chinese people of different ages in health promotion activities [39], future planning needs to review the efforts required and the potential effectiveness, as well as exploring strategies for engaging middle aged people and addressing research participation concerns in Chinese community. The follow-up rate of 61% was reasonable when compared with six-month retention rates in previously reported community-based preventative health programs [40, 41].

In this study, an improvement in HBV-related knowledge was observed in both groups, especially in relation to understanding routes of transmission, which has been reported to be a main knowledge gap in Chinese community [23, 41–43]. Following the education program, 12% of participants were tested, and another 15% of participants requested HBV tests from their doctors or confirmed their vaccination history. The effects of the intervention were comparable with previous community-based HBV-related education programs conducted in the US [36, 39, 41], though effects varied by particular strategy. Two studies reporting lay health worker-led home visit education program targeting Hmong Americans [36] and Chinese Americans and Canadians [39] showed an HBV testing rate of 24 and 15%, respectively. Notably in the study among Hmong Americans, half of those who reported being tested for HBV following an education session also received testing recommendation from their doctor, a factor shown in our study and others to be an important motivator for HBV testing [44]. Our study found higher HBV testing uptake in the liver cancer information arm (17%) compared with the standard information arm (7%). This difference was not statistically significant, and a larger study is required to reliably establish the potential impact of a liver cancer-based education program. While a full-scale randomised controlled study is required to answer which message works better, the present study suggested several barriers in the pathway of using an education program to enable individuals at risk to request a HBV test at their healthcare providers, which need to be addressed in larger planning efforts.

Key factors identified from this study affecting participants’ testing behaviour including risk perception, healthcare access issue, relationships with GPs, and contextual factors. Among participants who perceived a low risk of HBV infection, factors such as lack of symptoms, living in developed regions, or the belief that a good lifestyle prevents disease were used to justify their risk perception. This may relate to a persistent misunderstandings of HBV risk [23, 43] or generalised risk denial [45], where people rate the risks to themselves and the risks to their peers differently. In this case, individual-based risk assessment, rather than a health promotion program showing overall risk of HBV in targeting population, may help increase HBV testing rates.

Another barrier to HBV testing identified in this study was not knowing where to get tested and concern of costs, consistent with previous studies [10, 11] suggesting that health service access was one of the determinants of HBV testing uptake among Chinese-born Australian residents. Notably in this study, almost all participants who expressed concerns about healthcare access were from the young adult group. To minimise this barrier, a clear and unambiguous message informing people where and how to be tested for HBV, including the cost of testing for non-Australian citizens, is likely to help. This information is particularly needed in the young Chinese population who are frequently new residents in Australia, however studies are needed to explore this further.

Other factors that were identified, such as participants’ relationships with their GP and the call for an accessible program delivering tests to the community, suggested that additional support from healthcare providers and public health programs are needed. Most participants in our study were able to contact a GP, which could have been an enabler for HBV testing. While it is important to empower the community to initiate the HBV discussion with their GPs, there are opportunities if HBV testing being included in a health check-up package or if primary care providers actively perform HBV risk assessment and advise the testing [36]. To meet demands for easier-to-access HBV testing, solutions such as community-based testing programs using point-of-care tests or innovative sampling methods (such as using dried blood spot test which could be easily mailed for HBV testing at the ease of one’s home), might support at-risk population to engage in HBV testing [38, 46].

There were several limitations in the study. The findings of the pilot study, which primarily served the purpose of assessing feasibility and providing insights for a larger trial, need to be interpreted with caution. The small sample size and the bimodal age distribution of participants included in this pilot means our results should not be interpreted as representative of the broader Australian Chinese population. Eligibility assessment and outcome measures were largely based on self-report data, including reporting six months after the intervention, which might be susceptible to recall and response bias. Misclassification bias may also impact the results due to some participants not being aware of their vaccination status at the time of recruitment, however this occurred equally in both study arms so would have been unidirectional bias. Research was not core business for most of the community-based organisations/groups that provided space or opportunities for study recruitment; at several sites, only those who self-selected would present to fieldworkers for eligibility assessment, which might lead to selection bias. The time of follow up may have been too short to allow for opportunistic testing to occur. Although the comparison of demographics between people completed and did not complete follow-up did not suggest any difference, the outcomes might still subject to loss-to-follow-up bias given the small sample size. The follow-up rate and access to HBV testing in our study was likely impacted by international travel restrictions, restricted community health services access, economic stress and increased competing priorities for participants due to COVID-19 related lockdowns during the study follow-up period.

## Conclusion

This study evaluated feasibility, acceptability, and the impact of specific educational messages on HBV testing uptake in priority populations in Australia, and provided essential data to inform design of future intervention studies and education programs. Taken together, the study demonstrated that a tailored education program targeting ethic Chinese in Australia was feasible and acceptable to participants. Pilot results suggested HBV testing uptake remained suboptimal in both groups and a larger study is required to determine differences in effectiveness. Future health promotion programs need to be developed in partnership with targeting community and incorporate the relevance of HBV risks to the target audience, testing access and costs. In addition to education programs, support from healthcare providers is needed to increase HBV testing among Chinese born people, and alternative community-based testing programs such as using point-of-care tests may help reduce barriers for HBV testing.

## Supplementary Information

**Additional file 1: Figure s1.** Information sheet. **Table s1.** Recruitment at each site. **Table s2.** Hepatitis B-related questions asked at baseline and follow-up questionnaire. **Table s3** Semi-structured interview guide (after follow-up questionnaire). **Table s4.** Demographics of participants at baseline and follow-up; comparison of respondents and non-respondents of follow-up, overall and in each group. **Table s5.** Acceptability of resources and education program, and suggestions for improvement. **Table s6.** Knowledge change pre-and post-intervention. **Table s7.** Comparison of variables from follow-up questionnaire between participants in different age group (*n* = 33). **Table s8.** CONSORT 2010 Checklist of information to include when reporting a pilot or feasibility trial*. **Table s9** CONSORT 2010 checklist of information to include when reporting a pilot or feasibility randomized trial in a journal or conference abstract*. **Table s10.** COREQ (COnsolidated criteria for REporting Qualitative research) Checklist: 32-item checklist*.

## Data Availability

The datasets generated during and/or analysed during the current study are not publicly available due to ethics requirement but are available from the corresponding author on reasonable request.

## Abbreviations

HBV: hepatitis B virus
CHB: chronic hepatitis B
