# Supplementary material for: Assessing the feasibility, acceptability and impacts of an education program on hepatitis B testing uptake among ethnic Chinese in Australia: results of a randomised controlled pilot study
Source: BMC Public Health. 2021 Oct 15;21:1861. doi: 10.1186/s12889-021-11916-0 (PMC8518279; doi:10.1186/s12889-021-11916-0)
Supplement: Supplementary file 1 — Additional file 1: Figure s1. Information sheet. Table s1. Recruitment at each site. Table s2. Hepatitis B-related questions asked at baseline and follow-up questionnaire. Table s3 Semi-structured interview guide (after follow-up questionnaire). Table s4. Demographics of participants at baseline and follow-up; comparison of respondents and non-respondents of follow-up, overall and in each group. Table s5. Acceptability of resources and education program, and suggestions for improvement. Table s6. Knowledge change pre-and post-intervention. Table s7. Comparison of variables from follow-up questionnaire between participants in different age group (n = 33). Table s8. CONSORT 2010 Checklist of information to include when reporting a pilot or feasibility trial*. Table s9 CONSORT 2010 checklist of information to include when reporting a pilot or feasibility randomized trial in a journal or conference abstract*. Table s10. COREQ (COnsolidated criteria for REporting Qualitative research) Checklist: 32-item checklist*. [file 12889_2021_11916_MOESM1_ESM.docx]

# Supplementary materials

### Figure s1 Information sheet


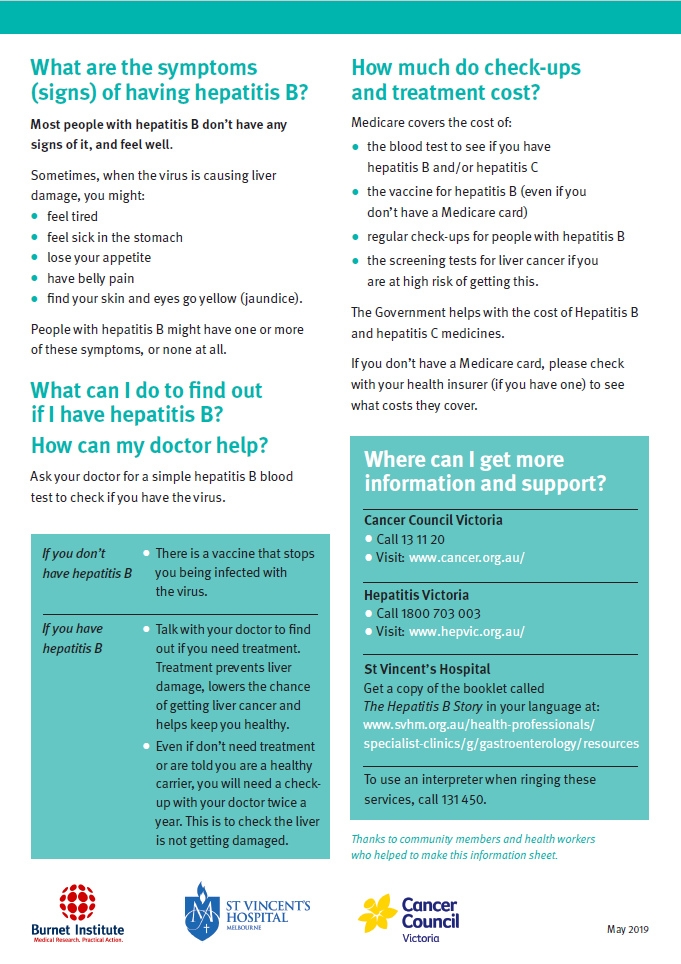

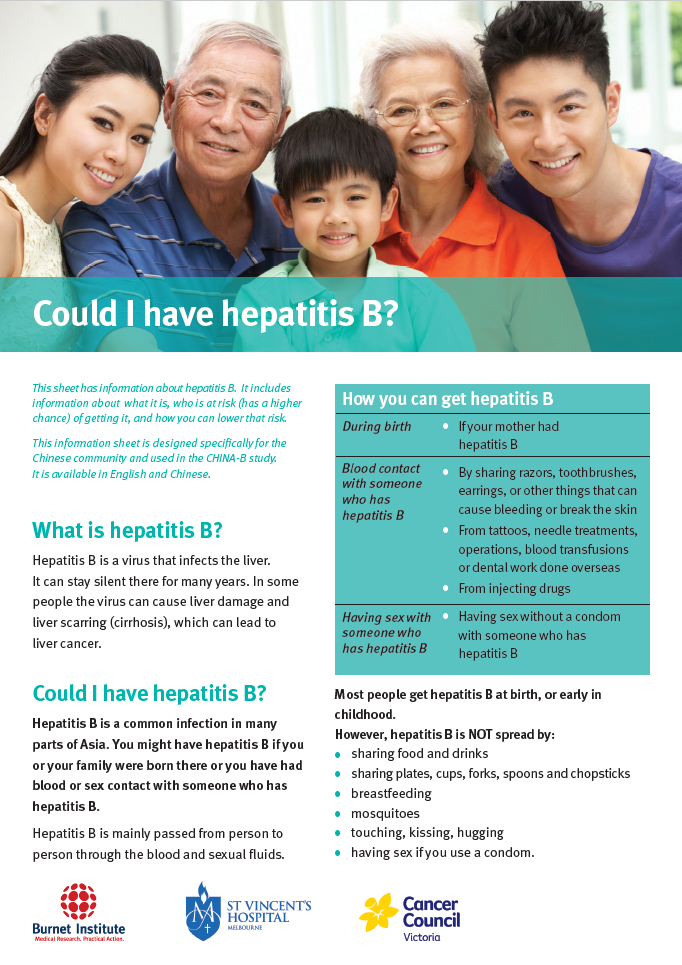


Arm 1

Hepatitis B-centred information, English version

Arm 2

Liver cancer prevention-centred information, English version


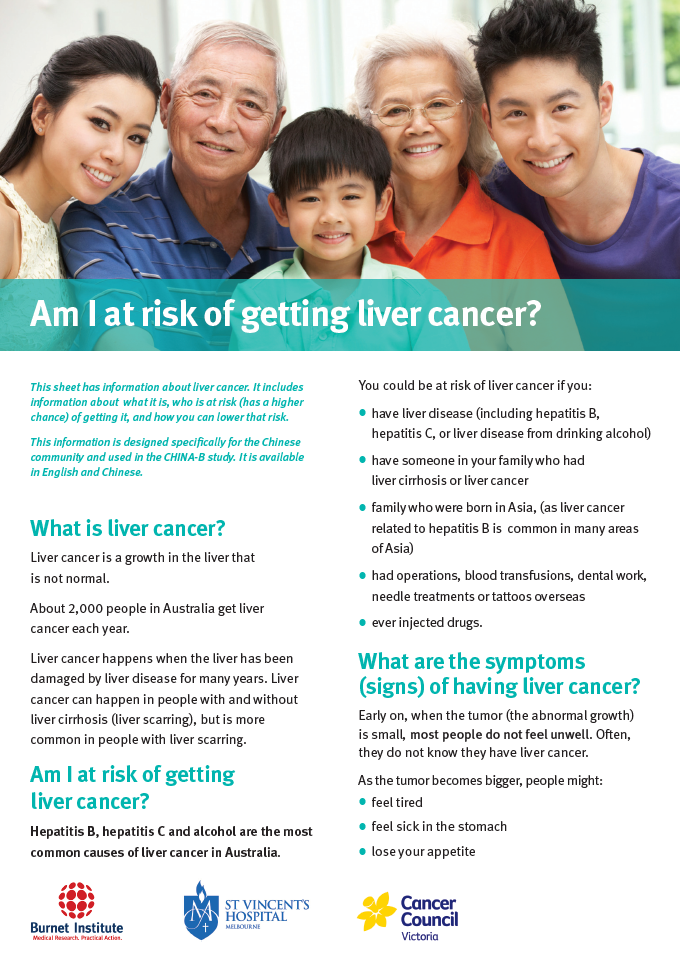

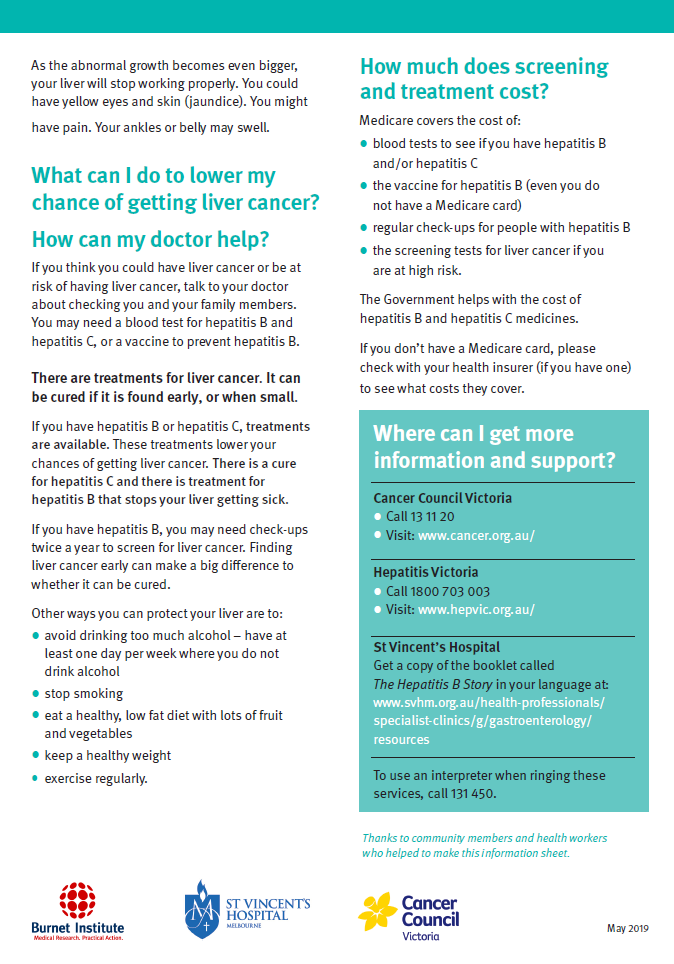


### Table s1 Recruitment at each site

| Site | Type of the site | Number of recruitment visits | Study recruitment | Number of people approached | Number of people assessed for eligibility | Number of people excluded | | | Number of people recruited |
| --- | --- | --- | --- | --- | --- | --- | --- | --- | --- |
|  |  |  |  |  |  | Ever tested or vaccinated | Not Chinese or cannot provide written consent | Declined |  |
| 1 | Community monthly immunisation session | 3 | People attending the site were screened eligibility and invited to study participation | 33 | 33 | 15 | 0 | 15 | 3 |
| 2 | Community health services | 7 | People attending the site were screened eligibility and invited to study participation | 36 | 36 | 15 | 6 | 7 | 8 |
| 3 | Local senior people association | 2 | Study promotion in the form of mini seminar | 60* | 12 | 4 | 0 | / | 8 |
| 4 | Local senior people association | 1 | Study promotion in the form of mini seminar | 20* | 1 | 1 | 0 | / | 0 |
| 5 | Community book reading club | 1 | Study promotion in the form of mini seminar | 15 | 1 | 0 | 0 | / | 1 |
| 6 | Community center weekly program | 1 | Study promotion in the form of mini seminar | 30* | 1 | 0 | 0 | / | 1 |
| 7 | Local senior people association | 1 | Study promotion in the form of mini seminar | 40* | 4 | 2 | 0 | / | 2 |
| 8 | Community event | 1 | Study flyer distributed; EOI form invited | 60* | 2 | 0 | 0 | / | 2 |
| 9 | Community festival | 1 | Study flyer distributed; EOI form invited | 25* | 0 | / | / | / | / |
| 10 | Community neighbourhood house | 1 | Poster with study information set up in the site one week prior to study recruitment | ^#^ | 0 | / | / | / | / |
|  | University campus | / | Online student bulletin promotion | ^#^ | 40 | 12 | 1 | 6 | 21 |
|  | Public venue, various | / | Online promotion and radio broadcast | ^#^ | 12 | 4 | 0 | / | 8 |
| * estimates. ^#^ cannot be estimated | | | | | | | | | |

### Table s2 Hepatitis B-related questions asked at baseline and follow-up questionnaire

| Q1. Do you know whether any of your family members are living with/infected/have hepatitis B? |
| --- |
| Q2. How do you think people get hepatitis B? |
| Q3. Do you think there is treatment available for hepatitis B? |
| Q4. What do you think hepatitis B can do to your body? |

### Table s3 Semi-structured interview guide (after follow-up questionnaire)

| Key aspects | Prompt questions |
| --- | --- |
| Understanding of hepatitis B | - Could you please tell me what you know about hepatitis B? |
| Understanding of HBV testing procedure | - How do you think you could be tested for hepatitis B? |
| Testing intention | - Have you considered getting a hepatitis B test since participating in the study? Why or why not? |
| Facilitators and barriers for HBV testing | - What are some of the reasons that you think that people in general would get hepatitis B testing? - What do you think is a barrier that stops people from getting tested? |
| Feedback on the information leaflet | - In regard to the leaflet that you received as part of the study, how did you find that? - What are some of the things you found useful? - Any things that could have been better about it? |
| Feedback on the education session | - Is there anything that could have been better about the education session? |

### Table s4 Demographics of participants at baseline and follow-up; comparison of respondents and non-respondents of follow-up, overall and in each group


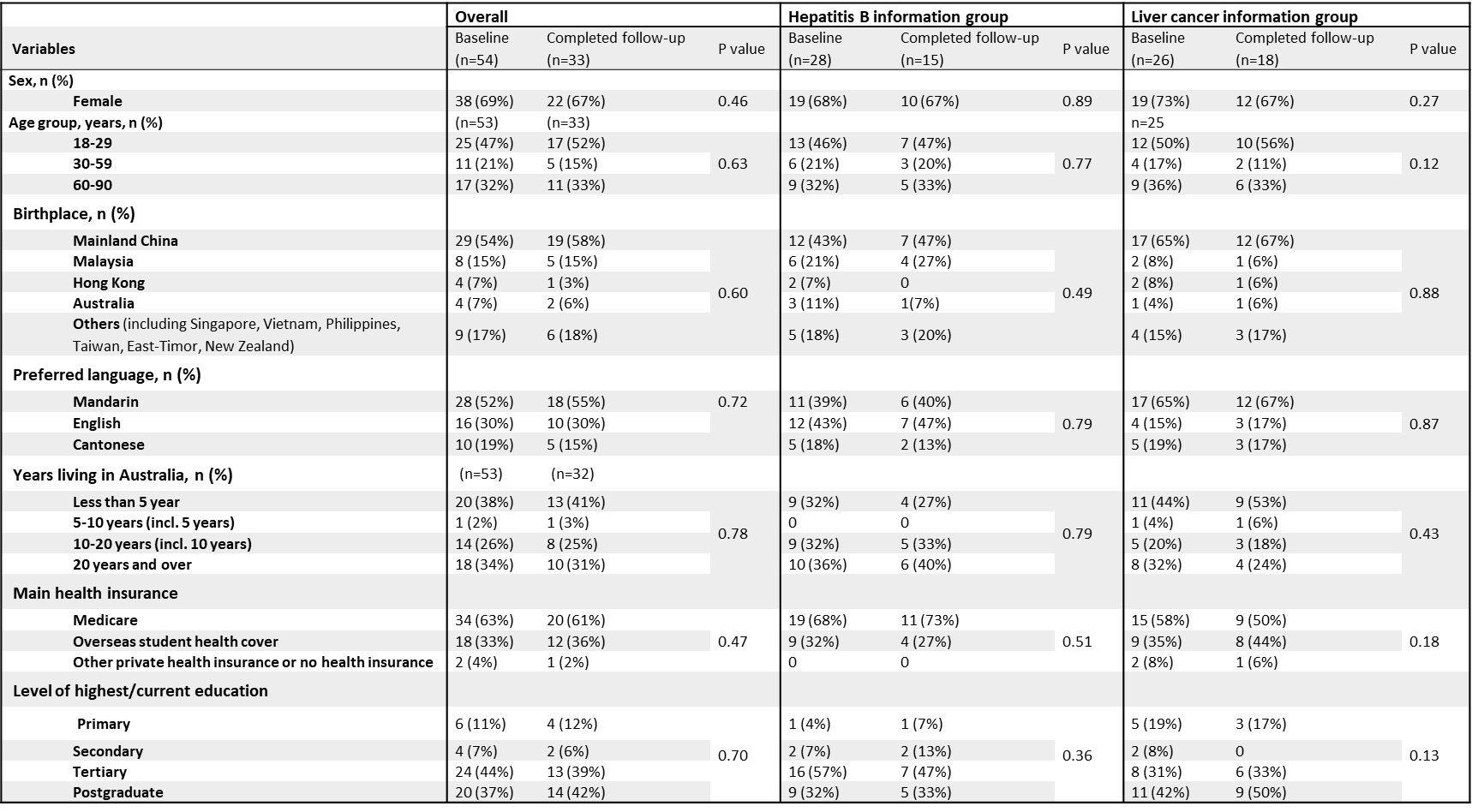


### Table s5 Acceptability of resources and education program, and suggestions for improvement

|  | **Arm 1**  **Hepatitis B information (n=15)** | **Arm 2**  **Liver cancer prevention information (n=18)** | **Overall**  **(n=33)** |
| --- | --- | --- | --- |
| Median score of agreement level on a scale of 0 to 5  (0- strongly disagree, 5- strongly agree) |  |  |  |
| The information leaflet is easy to understand. | 5 | 5 | 5 |
| The information provided in the leaflet is relevant to me. | 4 | 3 | 3 |
| The explanation provided by the researcher was easy to follow. | 5 | 5 | 5 |
| The explanation provided by the researcher helped me understand the information in the leaflet better. | 4.5 | 4 | 4 |
| Comments and suggestions representatives | *“I liked how it was easy to read and you know direct and to do the point and it wasn’t too heavy on the info.”*  *“it was pretty concise.”*  *“don’t really remember”*  *“Oral explanation is more important than the information leaflet. I have forgotten what’s in the leaflet, but I still remember what she told me”*  *“probably more visual things, like pictures, I think that would help” “I think it was more specific to Asian ethnicity and it explained briefly and in simple terms”* | *“I don’t think it contains so many difficult terminologies, just the normal language that other people will understand”*  *“Don’t remember the details”*  *“The disadvantage, like I said, that if you want to know more I don’t think it’s enough.”*  *“need to include treatment information”*  *“more information is needed about how to get vaccination”*  *“Emailing the resource, would be easier to use”* |  |

### Table s6 Knowledge change pre-and post-intervention

|  | **Arm 1**  **Hepatitis B information (n=15)** | | **Arm 2**  **Liver cancer prevention information (n=18)** | | **Arm 1**  **Percentage change (95% CI)** | **Arm 2**  **Percentage change (95% CI)** |
| --- | --- | --- | --- | --- | --- | --- |
|  | Before | After | Before | After |  |  |
| **Response to “How do you think you could get hepatitis B?”** |  |  |  |  |  |  |
| *Blood* | 8 | 9 | 4 | 10 | 7% (2%-32%) | 33% (13%-59%) |
| *Sexual* | 2 | 6 | 4 | 5 | 27% (8%-55%) | 6% (1%-27%) |
| *Mother- to-child* | 0 | 1 | 2 | 4 | 7% (2%-32%) | 11% (1%-35%) |
| *Saliva/sharing food/eating* (incorrect) | 8 | 4 | 11 | 4 | Reduced 27%  (8%-55%) | Reduced 39%  (17%-64%) |
| *Genetic/ inherited* (incorrect) | 3 | 1 | 2 | 2 | Reduced 13%  (2%-40%) | - |
| *Unsure/don’t know* | 1 | 2 | 4 | 1 | 7% (2%-32%) | Reduced 17% (4%-41%) |
| **Response to “Do you think there is treatment available for hepatitis B?”** |  |  |  |  |  |  |
| *Yes* and *very likely* | 10 | 11 | 10 | 11 | 7% (2%-32%) | 6% (1%-27%) |
| *No* and *unsure/don’t know* | 5 | 4 | 8 | 7 | Reduced 7% (2%-32%) | Reduced 6% (1%-27%) |

### Table s7 Comparison of variables from follow-up questionnaire between participants in different age group (n=33)

| Item | | Age group | | |
| --- | --- | --- | --- | --- |
|  |  | 18-29 years (n=17) | 30-59 years  (n=5) | 60-90 years  (n=11) |
| Self-reported hepatitis B-related knowledge change | Improved, largely | 1 | 1 | 0 |
|  | Improved, moderately/slightly | **15** | **4** | **2** |
|  | No improvement or neutral | 1 | 0 | **7** |
|  | Unsure | 0 | 0 | **2** |
| Visited a doctor in last six months | | **7** | **3** | **11** |
| Discussed about hepatitis B testing and get tested | | 1 | 0 | **3** |
| Discussed about hepatitis B testing but didn’t get tested | | 0 | 0 | **3** |
| Didn’t discuss hepatitis B testing | | **6** | **3** | **5** |
| Keywords comparisons (comments on not discussing hepatitis B testing with a GP or no intention of getting a hepatitis B test) | | *“never came into mind”*  *“don’t feel the need”*  *“not sure how”*  *“never saw a GP before”*  *“only went to GP for … (reason)”*  *“busy”*  *“lack of priority”*  *“COVID got in the way”* | *“never have any relevant symptoms”*  *“forgot”*  *“I think I did similar tests when coming to Australia”* | *“I don’t have hepatitis B”*  *“I don't ask for any tests from doctors; I'll do what doctor asks me to test”*  *“saw doctor for … (reasons)”*  *“telehealth for other diseases”* |

### Table s8 CONSORT 2010 Checklist of information to include when reporting a pilot or feasibility trial*

*Adapted from Eldridge SM, Chan CL, Campbell MJ, Bond CM, Hopewell S, Thabane L, et al. CONSORT 2010 statement: extension to randomised pilot and feasibility trials. BMJ. 2016;355.

| Section/Topic | Item No | Checklist item | Reported on page No |
| --- | --- | --- | --- |
| Title and abstract | | | |
|  | 1a | Identification as a pilot or feasibility randomised trial in the title | 1 |
|  | 1b | Structured summary of pilot trial design, methods, results, and conclusions (for specific guidance see CONSORT abstract extension for pilot trials) | 4-5 |
| Introduction | | | |
| Background and objectives | 2a | Scientific background and explanation of rationale for future definitive trial, and reasons for randomised pilot trial | 6-7 |
|  | 2b | Specific objectives or research questions for pilot trial | 7 |
| Methods | | | |
| Trial design | 3a | Description of pilot trial design (such as parallel, factorial) including allocation ratio | 8 |
|  | 3b | Important changes to methods after pilot trial commencement (such as eligibility criteria), with reasons | N/A |
| Participants | 4a | Eligibility criteria for participants | 8 |
|  | 4b | Settings and locations where the data were collected | 8-9 |
|  | 4c | How participants were identified and consented | 8-9 |
| Interventions | 5 | The interventions for each group with sufficient details to allow replication, including how and when they were actually administered | 9 |
| Outcomes | 6a | Completely defined prespecified assessments or measurements to address each pilot trial objective specified in 2b, including how and when they were assessed | 10-11 |
|  | 6b | Any changes to pilot trial assessments or measurements after the pilot trial commenced, with reasons | N/A |
|  | 6c | If applicable, prespecified criteria used to judge whether, or how, to proceed with future definitive trial | N/A |
| Sample size | 7a | Rationale for numbers in the pilot trial | 8 |
|  | 7b | When applicable, explanation of any interim analyses and stopping guidelines | N/A |
| Randomisation: |  |  |  |
| Sequence  generation | 8a | Method used to generate the random allocation sequence | 9 |
|  | 8b | Type of randomisation(s); details of any restriction (such as blocking and block size) | 9 |
| Allocation  concealment  mechanism | 9 | Mechanism used to implement the random allocation sequence (such as sequentially numbered containers), describing any steps taken to conceal the sequence until interventions were assigned | 9 |
| Implementation | 10 | Who generated the random allocation sequence, who enrolled participants, and who assigned participants to interventions | N/A |
| Blinding | 11a | If done, who was blinded after assignment to interventions (for example, participants, care providers, those assessing outcomes) and how | 9 |
|  | 11b | If relevant, description of the similarity of interventions | N/A |
| Statistical methods | 12 | Methods used to address each pilot trial objective whether qualitative or quantitative | 11 |
| Results | | | |
| Participant flow (a diagram is strongly recommended) | 13a | For each group, the numbers of participants who were approached and/or assessed for eligibility, randomly assigned, received intended treatment, and were assessed for each objective | 12; Figure 1 |
|  | 13b | For each group, losses and exclusions after randomisation, together with reasons | 12; Figure 1 |
| Recruitment | 14a | Dates defining the periods of recruitment and follow-up | 8 |
|  | 14b | Why the pilot trial ended or was stopped | N/A |
| Baseline data | 15 | A table showing baseline demographic and clinical characteristics for each group | Table 1 |
| Numbers analysed | 16 | For each objective, number of participants (denominator) included in each analysis. If relevant, these numbers  should be by randomised group | 13; Table 1 |
| Outcomes and estimation | 17 | For each objective, results including expressions of uncertainty (such as 95% confidence interval) for any estimates. If relevant, these results should be by randomised group | 13 (95%CI data removed as suggested by reviewers) |
| Ancillary analyses | 18 | Results of any other analyses performed that could be used to inform the future definitive trial | 13-17 |
| Harms | 19 | All important harms or unintended effects in each group (for specific guidance see CONSORT for harms) | N/A |
|  | 19a | If relevant, other important unintended consequences | N/A |
| Discussion | | | |
| Limitations | 20 | Pilot trial limitations, addressing sources of potential bias and remaining uncertainty about feasibility | 20-21 |
| Generalisability | 21 | Generalisability (applicability) of pilot trial methods and findings to future definitive trial and other studies | 17-20 |
| Interpretation | 22 | Interpretation consistent with pilot trial objectives and findings, balancing potential benefits and harms, and  considering other relevant evidence | 17-20 |
|  | 22a | Implications for progression from pilot to future definitive trial, including any proposed amendments | 18-19 |
| Other information | | |  |
| Registration | 23 | Registration number for pilot trial and name of trial registry | N/A |
| Protocol | 24 | Where the pilot trial protocol can be accessed, if available | N/A |
| Funding | 25 | Sources of funding and other support (such as supply of drugs), role of funders | Included with submission |
|  | 26 | Ethical approval or approval by research review committee, confirmed with reference number | 4 |

### Table s9 CONSORT 2010 checklist of information to include when reporting a pilot or feasibility randomized trial in a journal or conference abstract*

*Adapted from Eldridge SM, Chan CL, Campbell MJ, Bond CM, Hopewell S, Thabane L, et al. CONSORT 2010 statement: extension to randomised pilot and feasibility trials. BMJ. 2016;355.

| **Item** | **Description** | **Reported on line number** |
| --- | --- | --- |
| Title | Identification of study as randomised pilot or feasibility trial | √ |
| Trial design | Description of pilot trial design (eg, parallel, cluster) | 8 |
| Methods |  |  |
| Participants | Eligibility criteria for participants and the settings where the pilot trial was conducted | 8-10 |
| Interventions | Interventions intended for each group | 10-12 |
| Objective | Specific objectives of the pilot trial | 5-8 |
| Outcome | Prespecified assessment or measurement to address the pilot trial objectives** | 12-18 |
| Randomization | How participants were allocated to interventions | 10 |
| Blinding (masking) | Whether or not participants, care givers, and those assessing the outcomes were blinded to group assignment | N/A |
| Results |  |  |
| Numbers randomized | Number of participants screened and randomised to each group for the pilot trial objectives** | 19-20 |
| Numbers analysed | Number of participants analysed in each group for the pilot objectives** | 19-23 |
| Outcome | Results for the pilot objectives, including any expressions of uncertainty** | 20-26 |
| Harms | Important adverse events or side effects | N/A |
| Conclusions | General interpretation of the results of pilot trial and their implications for the future definitive trial | 27-32 |
| Trial registration | Registration number for pilot trial and name of trial register | N/A |
| Funding | Source of funding for pilot trial | N/A |
| **Space permitting, list all pilot trial objectives and give the results for each. Otherwise, report those that are a priori agreed as the most important to the decision to proceed with the future definitive RCT. | | |

### Table s10 COREQ (COnsolidated criteria for REporting Qualitative research) Checklist: 32-item checklist*

*Adapted from: Tong A, Sainsbury P, Craig J. Consolidated criteria for reporting qualitative research (COREQ): a 32-item checklist for interviews and focus groups. International Journal for Quality in Health Care. 2007. Volume 19, Number 6: pp. 349 – 357

| **Topic** | **Item No.** | **Guide Questions/Description** | **Reported on Page No. or detailed information if not reported** |
| --- | --- | --- | --- |
| **Domain 1: Research team and reflexivity** | | | |
| *Personal characteristics* | | | |
| Interviewer/facilitator | 1 | Which author/s conducted the interview or focus group? | YX, MA |
| Credentials | 2 | What were the researcher’s credentials? E.g., PhD, MD | YX has MBBS, MMed; MA has BBmed, Bsc(Hons) |
| Occupation | 3 | What was their occupation at the time of the study? | YX was a PhD student; MA was a research assistant |
| Gender | 4 | Was the researcher male or female? | Both female |
| Experience and training | 5 | What experience or training did the researcher have? | YX had medical training; both had public health research trainings and experiences conducting qualitative interviews |
| *Relationship with participants* | | |  |
| Relationship established | 6 | Was a relationship established prior to study commencement? | No |
| Participant knowledge of the interviewer | 7 | What did the participants know about the researcher? e.g., personal goals, reasons for doing the research | Participants knew the interviewers as public health researchers and reasons of the study |
| Interviewer characteristics | 8 | What characteristics were reported about the inter viewer/facilitator? e.g., Bias, assumptions, reasons and interests in the research topic | Reasons and interests in the research topic |
| **Domain 2: Study design** | | | |
| *Theoretical framework* | | | |
| Methodological orientation and Theory | 9 | What methodological orientation was stated to underpin the study? e.g. grounded theory, discourse analysis, ethnography, phenomenology, content analysis | Thematic analysis |
| *Participant selection* | | | |
| Sampling | 10 | How were participants selected? e.g., purposive, convenience, consecutive, snowball | Purpose sampling |
| Method of approach | 11 | How were participants approached? e.g., face-to-face, telephone, mail, email | Telephone |
| Sample size | 12 | How many participants were in the study? | Ten |
| Non-participation | 13 | How many people refused to participate or dropped out? Reasons? | Participants were self-opt-in the semi-structured interview; refusal was not recorded. |
| *Setting* | | | |
| Setting of data collection | 14 | Where was the data collected? e.g., home, clinic, workplace | Over phone |
| Presence of non-participants | 15 | Was anyone else present besides the participants and researchers? | NA |
| Description of sample | 16 | What are the important characteristics of the sample? e.g., demographic data, date | Described in Table 2. |
| *Data collection* | | | |
| Interview guide | 17 | Were questions, prompts, guides provided by the authors? Was it pilot tested? | Described in Table s3 |
| Repeat interviews | 18 | Were repeat inter views carried out? If yes, how many? | NA |
| Audio/visual recording | 19 | Did the research use audio or visual recording to collect the data? | Yes, reported on page 8 |
| Field notes | 20 | Were field notes made during and/or after the interview or focus group? | Yes, reported on page 7 |
| Duration | 21 | What was the duration of the interviews or focus group? | 15-30 minutes, reported on page 7 |
| Data saturation | 22 | Was data saturation discussed? | No |
| Transcripts returned | 23 | Were transcripts returned to participants for comment and/or correction? | NA |
| **Domain 3: analysis and findings** | | | |
| *Data analysis* | | | |
| Number of data coders | 24 | How many data coders coded the data? | One, reported on page 8 |
| Description of the coding tree | 25 | Did authors provide a description of the coding tree? | NA |
| Derivation of themes | 26 | Were themes identified in advance or derived from the data? | Derived from the data, reported on page 8 |
| Software | 27 | What software, if applicable, was used to manage the data? | NVivo 12 Plus, reported on page 8 |
| Participant checking | 28 | Did participants provide feedback on the findings? | NA |
| *Reporting* | | | |
| Quotations presented | 29 | Were participant quotations presented to illustrate the themes/findings? Was each quotation identified? e.g., participant number | Yes, reported on page 10-12 |
| Data and findings consistent | 30 | Was there consistency between the data presented and the findings? | Yes, page7, 10-12 and table s3 |
| Clarity of major themes | 31 | Were major themes clearly presented in the findings? | Yes, page 10-12 |
| Clarity of minor themes | 32 | Is there a description of diverse cases or discussion of minor themes? | NA |
